# Early outcomes of transverse perineal support in obstructed defecation syndrome with pathological perineal descent; a prospective cohort study

**DOI:** 10.1186/s12893-026-03888-y

**Published:** 2026-06-06

**Authors:** Haitham M. Azmy Basiouny, Ali Ahmed Shafik, Mohamed Yehia Elbarmelgi, Osama Refaie, Mohamed Tamer, Mahmoud Adel Abu Alwafa, Ahmed Mohamed Abdelaal

**Affiliations:** Cairo University, Cairo, Egypt

**Keywords:** Obstructed defecation, Pathological perineal descent, Transverse perineal support, Rectal intussusception

## Abstract

**Background:**

While current surgical approaches for obstructed defecation syndrome (ODS) primarily target rectocele and rectal intussusception, excessive perineal descent (PD) may contribute to persistent symptoms and suboptimal surgical outcomes. This study aimed to evaluate early outcomes of transverse perineal support (TPS), a procedure designed to reinforce the perineum, in patients with ODS and pathological PD.

**Patients and method:**

All consecutive patients with ODS and pathological PD were assessed for eligibility. After at least one year of conservative management (dietary fiber, biofeedback, laxatives) that failed to relieve symptoms, twenty-nine patients were evaluated preoperatively using the Cleveland Clinic Constipation Score (CCCS), Pelvic Organ Prolapse/Urinary Incontinence Sexual Questionnaire (PISQ-12), perineometry, anorectal manometry, and MRI defecography. All patients underwent TPS procedure and were followed up for a minimum of 6 months. The primary outcome was the change in CCCS at 6 months.

**Results:**

Significant postoperative improvements were observed. The CCCS decreased significantly, with a mean difference (Post - Pre) of -5.31 (95% CI: -6.13 to -4.48 ; *p* < 0.001). Postoperative PISQ-12 scores were significantly higher than preoperative scores, with median (IQR) values of 35 (34–36) and 25 (24–27), respectively (*p* = 0.003). Intrarectal pressure during push increased from 34.34 ± 6.03 mmHg preoperatively to 41.17 ± 5.85 mmHg postoperatively with a mean difference (Post - Pre) of 6.83 (95% CI: 5.41 to 8.24; *p* < 0.001). Perineal descent measured by perineometry decreased from 2.29 ± 0.55 cm preoperatively to 1.74 ± 0.51 cm postoperatively with a mean difference of -0.64 cm (95% CI: -0.75 to -0.52; *p* < 0.001). No mesh-related complications (erosion, chronic pain) were observed. Nine patients (31.03%) reported persistent symptoms. All had rectal intussusception. Compared with patients who improved, these patients were older, had smaller perineal bodies, and greater preoperative perineal descent.

**Conclusions:**

Transverse perineal support procedure may be an effective option for selected patients with ODS and pathological PD. Outcomes were less favorable in patients with rectal intussusception, older age (observed as an association only, not causation) or smaller perineal body dimensions, underscoring the importance of careful patient selection and individualized surgical planning. Given the small sample size and the absence of a control group, these findings should be interpreted with caution as associational rather than causal.

## Introduction

ODS is exhibited by fragmented stools, straining with defecation, tenesmus, feeling of incomplete emptying, self-digitation and urgency [1]. In ODS patients, colonic transit time is usually normal, but transit in the rectosigmoid region is delayed. ODS patients may have megarectum, large rectocele, rectal prolapse, enterocele, descending perineum or anismus [2].

The excessive straining is one of the main causes of PD. It leads to weakness of the pelvic floor muscles causing further straining and producing a vicious cycle [3]. PD has been reported in about 75–84% of patients complaining of ODS [4, 5], and a clear association exists between constipation and pathological perineal descent [6, 7]. Current surgical interventions for ODS primarily aim to correct rectocele and/or rectal intussusception [8, 9].

Superficial and deep transverse perineal muscles have a crucial role in prevention of pathological PD. They support the pelvic floor during defecation and provide protection for the perineum against high pressure the produced during straining, which, if unduly repetitive, will lead to weakness, and sagging of the perineal muscles that may lead to perineocele, enterocele and sigmoidocele [10].

Renzi et al. described transverse perineal support procedure as surgical intervention for excessive perineal descent in patients complaining of obstructed defecation syndrome. The procedure involved positioning a porcine dermal implant above the perineal superficial fascia and suturing it to the periosteum of the ischial tuberosities at the level of the superficial transverse perineal muscle insertion [11]. Although TPS procedure has been proposed as a surgical option, published data on its clinical outcomes are very limited. This study aimed to assess early clinical outcomes of TPS procedure using polypropylene mesh in patients with obstructed defecation syndrome and pathological perineal descent.

## Materials and methods

### Study type

This is prospective cohort study evaluating early outcomes of TPS procedure using polypropylene mesh in ODS with pathological PD. All consecutive patients presenting with ODS and pathological PD during the study period were assessed for eligibility. This was an exploratory study and The sample size was based on feasibility and consecutive enrollment during the 26-months study period.

*The primary objective* was to evaluate the change in the Cleveland Clinic Constipation Score (CCCS) at 6 months following TPS with polypropylene mesh in patients presented with ODS with pathological PD. *Secondary objectives* included assessment of operative time, postoperative pain intensity, hospital stay, incidence of surgical site infection, and other postoperative complications (including mesh erosion and chronic pain).

### Study settings

This study was conducted at Cairo University Hospitals over a 26-months period, from July 2023 to September 2025.

### Inclusion criteria

Patients presenting with obstructed defecation associated with pathological PD (descent of Anorectal Junction (ARJ) more than 3 cm below the Pubococcygeal line (PCL) on MRI defecography) were included *(In normal subjects during defecation*,* the anorectal junction descends 1–3 cm below pubococcygeal line)* [12]. All patients had failed at least a one year course of conservative management (dietary fiber, biofeedback therapy, and osmotic laxatives) prior to enrollment. Patients were fit for anesthesia and surgical procedure.

### Exclusion criteria included

1. Patients with psychological instability
2. Any substance addiction
3. Previous surgery for perineal descent syndrome
4. Connective tissue disease
5. Patients with neurological deficit
6. Uncontrolled DM
7. Previous rectal surgeries
8. Inability for lifestyle change post-operatively
9. Mentally disabled patient
10. Previous levator ani muscle injury
11. Patients with paraplegia

#### Patient enrollment process (Fig. 1)

During the study period, 55 patients were assessed for eligibility. Of these, 17 were excluded for not meeting inclusion criteria, with the following specific reasons: Psychological instability (*n* = 2), Connective tissue disease (*n* = 2), Neurological deficit (*n* = 2), Uncontrolled diabetes mellitus (*n* = 3), Previous rectal surgeries (*n* = 5), Mentally disabled patient (*n* = 2), and Previous levator ani muscle injury (*n* = 1). Of the 38 patients who met inclusion criteria and underwent the TPS procedure, 9 were lost to follow-up before completing the 6-months assessment. The remaining 29 patients completed the 6-month follow-up and were included in the final analysis.

**Fig. 1:**
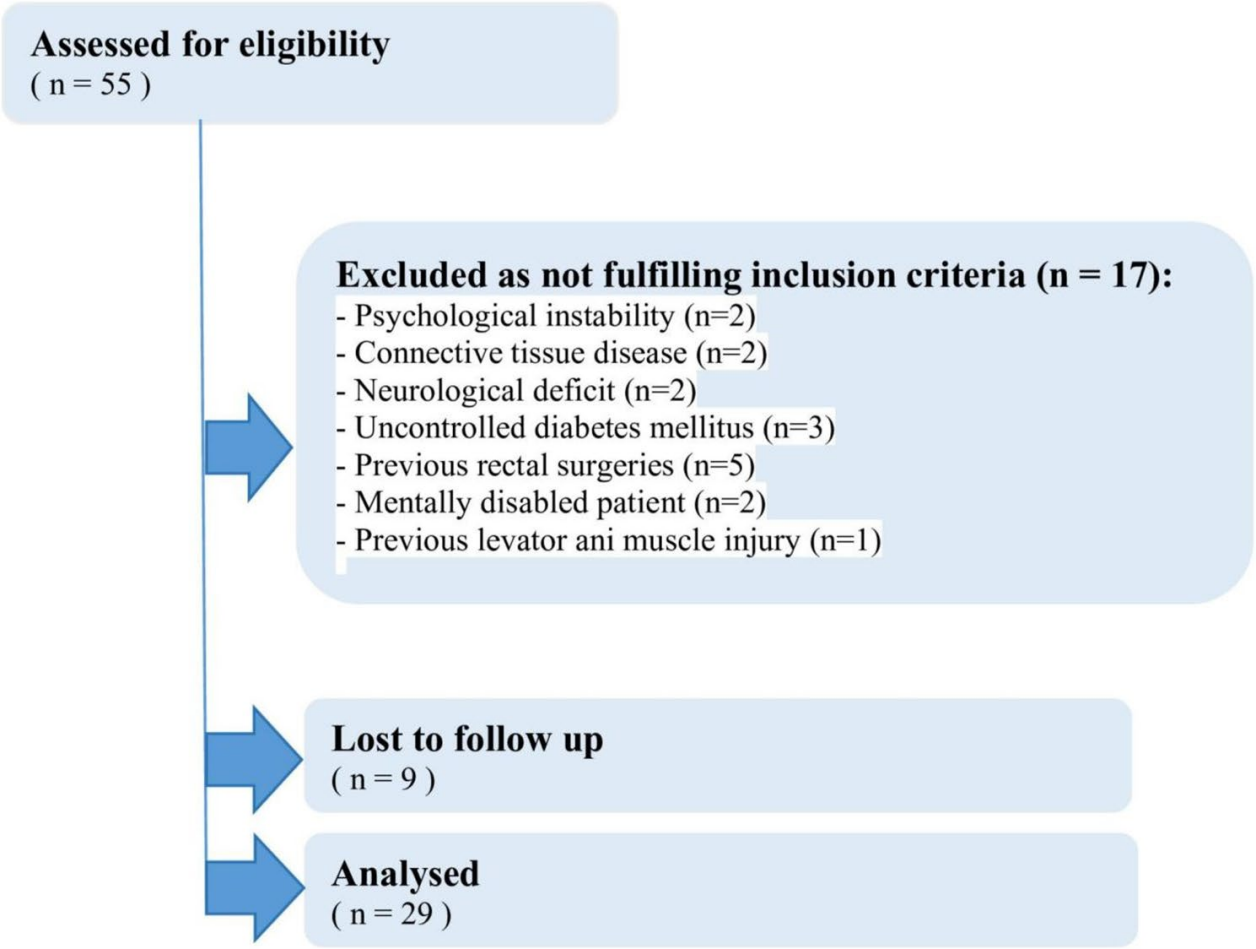
Patients flow chart

### Interventions

All eligible patients received detailed information about the study purpose, procedures, potential risks and benefits, and alternative treatment options. Written informed consent was obtained from all patients prior to enrollment. Patients were explicitly informed that participation was voluntary and that they could withdraw at any time without affecting their subsequent medical care. All patients also provided written consent for the surgical procedure itself. All 29 included patients underwent TPS using a polypropylene mesh. Polypropylene was chosen over the porcine dermal implant due to its lower cost, widespread availability, durability, and well-established safety profile in pelvic floor surgery. Preoperative assessment included a detailed history (age, disease duration, comorbidities, and prior surgical procedures), physical examination including digital rectal examination and body mass index (BMI), routine laboratory tests, psychological evaluation, dietary counseling, CCCS evaluation [13] and PISQ-12 questionnaire [14] for sexually active females and accepting to fulfill the questionnaire. Measurement of perineal descent was performed using perineometer, and anorectal manometry was performed in all patients. Preoperative Dynamic MRI Defecography was done. All MRI defecography studies were reported by a single senior radiologist with 8 years of experience in pelvic floor imaging. Intra-observer variability was not formally assessed. Postoperative follow-up was conducted for at least 6 months.

#### Perineometer measurement

**Fig. 2:**
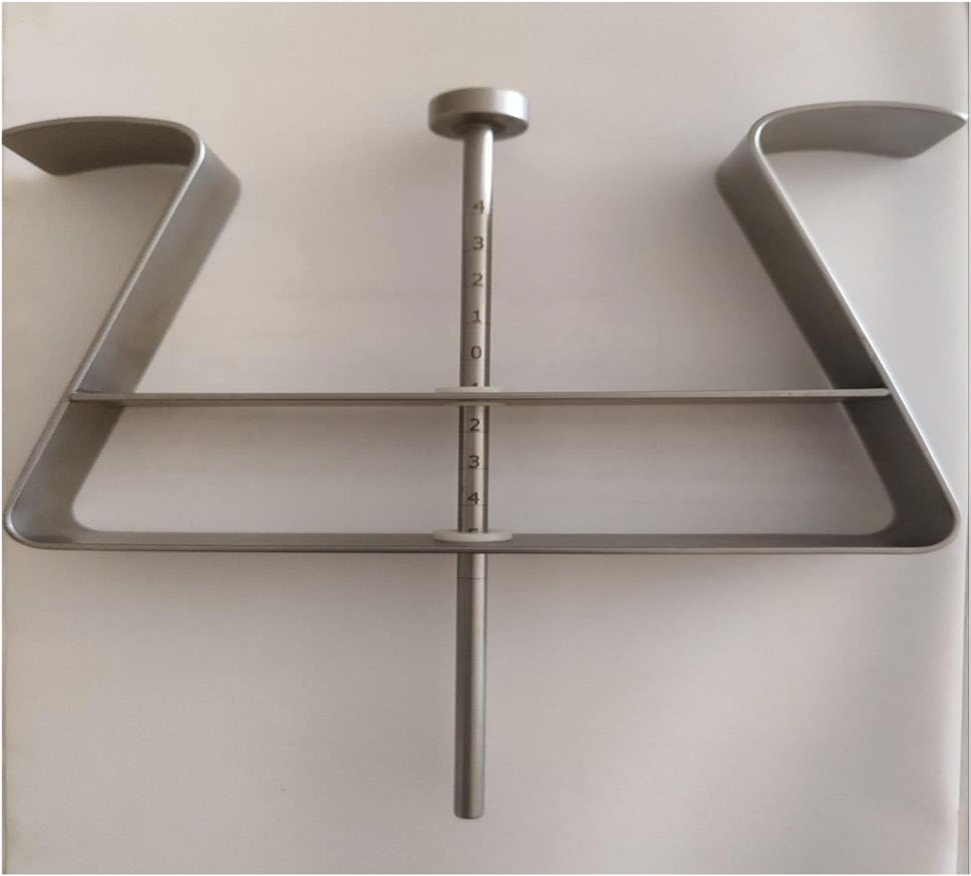
The Perineometer used for measurement of extent of perineal descent

(Figure 2) consists of a central cylinder with a centimeter scale that moves vertically within a stainless -steel frame, which has two limbs with transverse plates. With the patients in the left lateral position, the two stainless steel transverse plates were placed on both ischial tuberosities, and the central cylinder was positioned in contact with the anal verge, representing the perineum. The location of the perineum at rest was measured using the scale on the cylinder in relation to the plane of the ischial tuberosities. Patients were then instructed to bear down (straining, simulating the act of defecation), and the new location of the perineum was measured. The difference between the two measurements indicated the extent of perineal descent (in cm). All perineometer assessments were conducted by a single trained investigator to eliminate inter-rater variability. Each measurement was repeated three times within the same session, and the average value was recorded for analysis.

#### Anorectal manometry (ARM) protocol

Anorectal manometry was performed using a water-perfused catheter system with the patient in the left lateral position. Resting anal pressure was recorded for 5 min. Maximum squeeze pressure was measured by asking the patient to squeeze the anal canal as tightly as possible for 10 s. The push maneuver (simulated defecation) was performed by asking the patient to bear down as if to defecate. Intrarectal pressure during push was recorded as the maximum increase from baseline.

### Surgical procedure

**Fig. 3:**
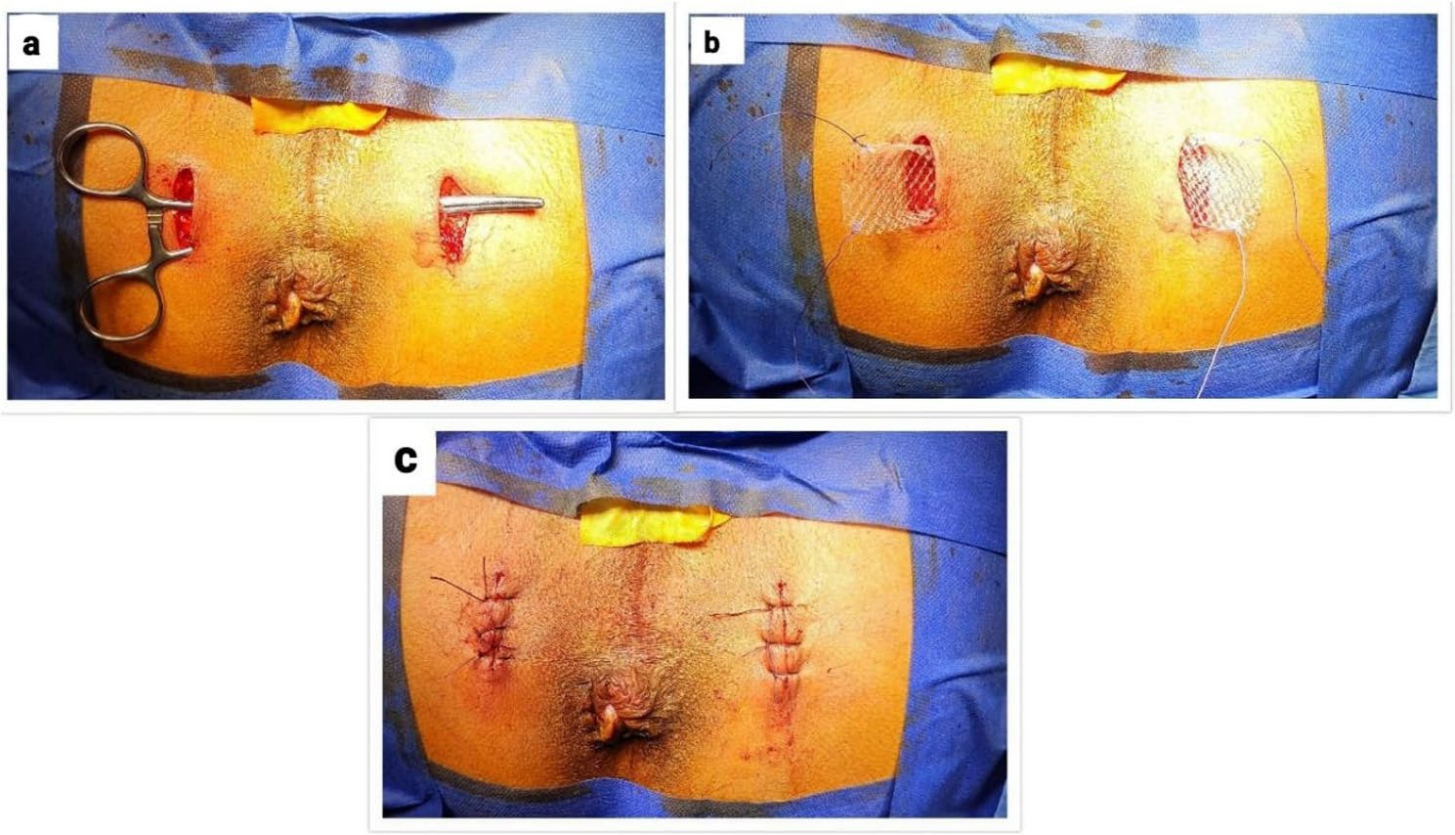
Transverse Perineal support procedure in a male patient: (**a**) Tunnel creation anterior to the superficial transverse perineal muscle by blunt dissection. **b** Placement and fixation of a polypropylene mesh to the ischial tuberosities. **c** Trimming of excess mesh and closure of skin

**Fig. 4:**
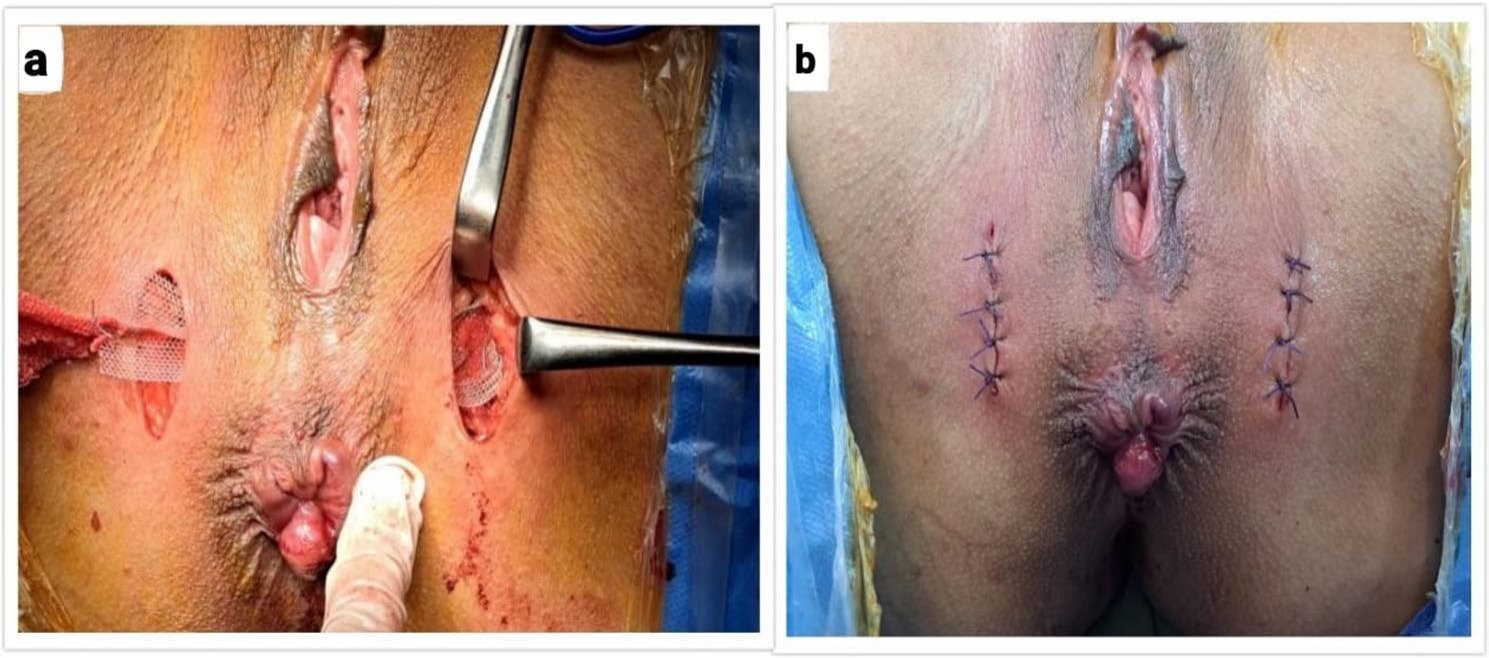
Transverse Perineal support procedure in a female patient: (**a**) Tunnel creation with mesh placement and fixation to ischial tuberosities. **b **Mesh trimming and skin closure

Patients received prophylactic antibiotics (metronidazole 500 mg IV and cefotaxime 1 gm IV) one hour before surgery. TPS procedure was conducted under spinal or general anesthesia in lithotomy position. All procedures were performed by a single colorectal surgeon with over 10 years of experience in pelvic floor surgery. The ischial tuberosities were identified, and bilateral skin incisions of approximately 2–2.5 cm were made over both ischial tuberosities. Blunt dissection anterior to the superficial transverse perineal muscle was done to create a tunnel, through which forceps were passed (Fig. 3a). A polypropylene synthetic mesh, measuring 3 × 11 cm, was tailored and positioned just above the transverse perineal muscle (Figs. 3b and 4a). The mesh was sutured to the periosteal membrane of both ischial tuberosities using polypropylene 2 − 0 sutures. Trimming of the excess mesh was done, and the skin incisions were sutured with vicryl 2 − 0 (Figs. 3c and 4b).

### Follow up

Patients were followed for a minimum of six months postoperatively, with monthly clinic visits during this period. Follow-up assessments included hospital stay, and complications such as surgical site infection, urinary retention, mesh erosion, and chronic groin or perineal pain. Pain intensity was assessed by Visual Analogue Scale (VAS), where 0–1 = no pain, 1.1–3 = low pain intensity, 3.1–7 = pain of medium intensity, 7.1–9 = pain of high intensity, and 9.1–10 = strong and unbearable pain [15]. The effect of the surgical intervention on obstructed defecation was evaluated using the CCCS. Additionally, the PISQ-12 questionnaire was reassessed in sexually active females who accepted and consented to fulfill the questionnaire. Anorectal manometry and perineometry were performed at six months postoperatively. Postoperative MRI defecography was not performed due to logistical constraints and patient burden.

### Statistical analysis

Statistical analyses were performed using SPSS version 28 (IBM Corp., Armonk, NY, USA). Continuous variables were reported as means and standard deviations (SD) when normally distributed and as median with interquartile range (IQR) when non-normally distributed. Categorical variables were reported as frequencies and percentages. Normality of paired differences was assessed using the Shapiro–Wilk test and histograms. For variables with normally distributed differences, paired-samples t-tests were used to compare preoperative and postoperative measurements, and mean differences were reported with 95% confidence intervals (CI). When normality assumptions were violated, Wilcoxon signed-rank test was applied. Differences between independent groups were assessed with the independent-samples (unpaired) t-test, with results reported as mean differences and corresponding 95% CIs [16]. Categorical variables were compared using Chi-square test, and Fisher’s exact test was used instead when the expected frequency is less than 5 [17]. Given the exploratory nature of this study and the small sample size, no adjustment for multiple comparisons was applied. All subgroup analyses are considered hypothesis-generating, and the results should be interpreted with caution regarding the risk of type I and type II errors. All statistical tests were two-tailed, and a P value < 0.05 was considered statistically significant.

## Results

### Demographic characteristics and preoperative MRI defecography findings

Of the 29 patients, 20 (68.96%) were females and 9 (31.04%) were males, with a mean age of 43.17 ± 4.59 years. All patients demonstrated the presence of rectocele in MRI, with a mean size of 2.5 ± 0.90 cm; 15 patients (51.72%) had grade I rectocele, and 14 (48.28%) had grade II. Intussusception was observed in 12 patients (41.37%), and 23 patients (79.31%) had grade I cystocele (Table 1).

**Table 1:** Demographic characteristics and preoperative MRI defecography findings

*n* = 29		
Age (years)	Mean	43.17
	SD^a^	± 4.59
Gender (n,%)	Females	20 (68.96%)
	Males	9 (31.04%)
BMI^b^	Mean	29.03
	SD	± 3.11
ASA grade (n,%)	ASA I	18 (62.07%)
	ASA II	10 (34.48%)
	ASA III	1 (3.45%)
HTN (n,%)		8 (27.58%)
DM (n,%)		6 (20.68%)
Cystocele in MRI (n,%)		23 (79.31%)
Preoperative ARJ descent in MRI (cm below PCL)^c^	Mean	6.82
	SD	± 1.78
Preoperative Rectocele in MRI (n,%)	Grade I	15 (51.72%)
	Grade II	14 (48.28%)
Preoperative Intussusception in MRI (n,%)	12 (41.37%)	

^a^Standard deviation

^b^Body Mass Index

^c^Pubo-cooccygeal line

### Operative time, post-operative pain and post-operative complications

The mean operative time was 43.21 ± 8.90 min. All patients were discharged 24 h postoperatively. The mean postoperative pain intensity score, assessed using VAS, was 4.45 ± 1.12. One patient developed surgical site infection at an incision over the ischial tuberosity, which was managed conservatively. No mesh-related complications (e.g., erosion, chronic perineal or groin pain) were observed during the 6-month follow-up period. No other complications, such as urinary retention, were observed.

### Postoperative changes in CCCS, perineal descent measured by perineometer and manometric findings

Paired-samples t tests were used to compare preoperative and postoperative values of the CCCS, perineal descent, and anorectal manometric parameters. There was a significant decrease in CCCS from preoperative to postoperative assessment, with a mean difference (Post - Pre) of −5.31 (95% CI: −6.13 to −4.48; *p* < 0.001). Extent of perineal descent measured by perineometry decreased postoperatively compared with preoperative measurements. Anorectal manometry revealed a significant increase in intrarectal pressure during push, from 34.34 ± 6.03 mmHg preoperatively to 41.17 ± 5.85 mmHg postoperatively, corresponding to a mean difference (Post - Pre) of 6.83 (95% CI: 5.41 to 8.24; *p* < 0.001). Other manometric parameters did not differ significantly from preoperative values. (Table 2).

**Table 2:** Postoperative changes in CCCS, perineometery, and manometric findings

*n* = 29	Preoperative		Postoperative		Mean Difference^b^ (95% CI^c^)	*P* value
	Mean	SD^a^	Mean	SD		
CCCS	18.00	3.21	12.69	4.65	−5.31 (−6.13,−4.48)	***P*** < 0.001*
Anal verge level (cm) during rest By perineometery (Above ischial tuberosity plane)	0.50	0.31	0.65	0.29	0.15 (0.23, 0.063)	***P*** **<** 0.001*
Anal verge level (cm) during straining By perineometery (Below ischial tuberosity plane)	1.79	0.82	1.01	0.86	−0.78 (−0.96,−0.61)	***P*** < 0.001*
Extent of the perineal descent By perineometery	2.29	0.55	1.66	0.63	−0.64 (−0.75,−0.52)	***P*** < 0.001^*^
Anal pressure during rest (in mm Hg)	69.45	9.41	68.55	7.02	−0.9 (−2.24,0.44)	*P =* 0.183
Anal pressure during squeeze (in mm Hg)	139.03	13.40	137.93	7.91	−1.1 (−4.85, 2.64)	*P =* 0.551
Intrarectal pressure during rest (in mm Hg)	12.31	3.31	11.66	2.65	−0.65 (−1.37, 0.06)	*P =* 0.073
Intrarectal pressure during push (in mm Hg)	34.34	6.03	41.17	5.85	6.83 (5.41, 8.24)	***P*** < 0.001^*^

^a^Standard deviation

^b^Mean difference calculated as Preoperative value minus Postoperative value

^c^Confidence interval

^d^Paired-samples t-test

^e^Interquartile range

^*^Statistically significant value

Persistent symptoms was observed in nine patients, 6 females (66.7%) and 3 males (33.3%), with no postoperative improvement in constipation scores.

### Post operative change in sexual function

Of the total study population, sixteen women were sexually active. Five of these patients refused to fulfill the PISQ-12 questionnaire. Consequently, PISQ-12 data were available for 11 women. This introduces a risk of responder bias. The sexual function analysis was exploratory. Comparison of preoperative and postoperative PISQ-12 scores using Wilcoxon signed-rank test showed a statistically significant improvement in sexual function after surgery. The median (IQR) PISQ-12 score increased from 25 (24–27) preoperatively to 35 (34–36) postoperatively (Wilcoxon signed-rank test, Z = − 2.956, *p* = 0.003) (Table 3).

**Table 3:** Postoperative changes in sexual function assessed by PISQ-12 questionnaire score

*n* = 11	Preoperative	Postoperative	Z^b^	*P* value
PISQ-12 score (Median, IQR)^a^	25 (24–27)	35 (34–36)	− 2.956	***P =*** 0.003^*^

^a^Interquartile range

^b^Standardized Wilcoxon signed-rank test statistic

^*^Statistically significant value

### Association between preoperative anorectal junction (ARJ) descent on MRI defecography and patient subgroups

Independent-samples *t* tests were used to compare preoperative anorectal junction descent on MRI defecography according to age group, presence of rectal intussusception and presence of rectocele. Patients aged > 40 years demonstrated significantly greater anorectal junction descent compared with those aged 40 years or younger (*p* = 0.035). Similarly, patients with rectal intussusception exhibited significantly greater perineal descent than those without intussusception (*p* = 0.006). In contrast, no significant difference in perineal descent was observed between patients with or without rectocele (*p* = 0.937) (Table 4).

**Table 4:** Association between preoperative anorectal junction (ARJ) descent on MRI defecography and patient subgroups

		Preoperative ARJ descent in MRI (cm below PCL)		Mean Difference (95% CI^b^)	*P* value
		Mean	SD^a^		
Age	> 40 years (*n = 14)*	6.82	1.78	1.16 (0.09, 2.23)	***P =*** 0.035^*^
	<=40 years (*n = 15)*	5.66	0.66		
Preoperative Rectocele	Grade I (*n = 15)*	6.20	1.56	−0.04 (−1.15, 1.06)	*P =* 0.937
	Grade II (*n = 14)*	6.24	1.34		
Preoperative Intussusception	Present (*n = 12)*	7.20	1.70	1.67 (0.76, 2.58)	***P =*** 0.006^*^
	No (*n = 17)*	5.53	0.60		

^a^Standard deviation

^b^Confidence interval

^*^Statistically significant value

### Preoperative perineal descent measurements obtained by Perineometry versus MRI defecography

Paired-samples *t* tests were used to compare perineal descent measurements obtained by MRI defecography and perineometry. Perineal descent was significantly underestimated when assessed using perineometry compared with MRI defecography. The mean difference (MRI - perineometry) was 3.93 cm (95% CI: 3.42 to 4.43; *p* < 0.001) (Table 5).

**Table 5:** Preoperative perineal descent measurements obtained by Perineometry versus MRI defecography

*n* = 29	Perineometer		MRI Defecography		Mean Differnce^b^ (95% CI^c^)	*P* value
	Mean	SD^a^	Mean	SD		
Preoperative perineal descent (in cm)	2.29	0.55	6.22	1.43	3.93 (3.42, 4.43)	***P*** < 0.001^*^

^a^Standard deviation

^b^Mean difference calculated as MRI value minus Perineometer value

^c^Confidence interval

^*^Statistically significant value

### Analysis of patients with persistent symptoms: (Tables 6 and 7)

**Table 6:** Analysis of patients with persistent symptoms (Part A)

	Persistent symptoms				Mean Difference (95% CI^b^)	*P* value
	Yes **(*n = 9)***		No **(*n = 20)***			
	Mean	SD^a^	Mean	SD		
Age (years)	55.67	7.05	37.55	6.38	18.12 (12.96, 23.54)	***P*** < 0.001^*^
Pre operative constipation score	21.33	0.50	16.50	2.72	4.83 (3.52, 6.14)	***P*** < 0.001*
Post operative constipation score	19.00	0.87	9.85	2.06	9.15 (8.03, 10.26)	***P*** **< 0.001** ^*^
Pre operative extent of perineal descent by perineometer (in cm)	2.67	0.13	2.12	0.59	0.55 (0.25, 0.82)	***P*** **< 0.001** ^*^
Post operative extent of perineal descent by perineometer (in cm)	2.33	0.25	1.47	0.34	0.86 (0.60, 1.12)	***P*** **< 0.001** ^*^
Perineal body cranio- caudal length (in mm)	16.67	1.04	18.32	0.72	−1.65 (−2.33, −0.96)	***P*** < 0.001^*^
Perineal body AP^c^ length (in mm)	14.70	0.15	15.74	0.62	−1.04 (−1.34, −0.73)	***P*** **< 0.001** ^*^
Preoperative ARJ^d^ descent below PC line^e^ in MRI (in cm)	7.50	1.89	5.65	0.62	1.85 (0.39, 3.31)	***P*** = 0.019^*^
Preoperative Rectocele in MRI (in cm)	2.50	0.90	2.50	0.92	0.00 (−0.75, 0.75)	*P* = 1
Postoperative Anal pressure during rest (in mm Hg)	62.33	2.17	71.35	2.17	−9.01(−13.7, −4.32)	***P =*** 0.001^*^
Postoperative Anal pressure during squeeze (in mm Hg)	136.0	4.58	138.3	8.99	−2.3 (−9.34, 3.74)	*P* = 0.388
Postoperative Intrarectal pressure during rest (in mm Hg)	13.66	3.27	10.75	1.74	2.91 (1.01, 4.81)	***P =*** 0.004^*^
Postoperative Intrarectal pressure during push (in mm Hg)	39.66	3.9	41.85	6.15	−2.18 (−7.01, 2.64)	*P* = 0.362

^a^Standard deviation

^b^Confidence interval

^c^Antero-Posterior

^d^Ano-Rectal Junction

^*^Statistically significant value

**Table 7:** Analysis of patients with persistent symptoms (Part B)

		Persistent symptoms				(X^2^)^a^	*P* value
		Yes **(*n = 9)***		No **(*n = 20)***			
		Count	%	Count	%		
Gender	Male	3	33.33%	6	30.0%	0.032	*P* = 1
	Female	6	66.67%	14	70.0%		
Cystocele in MRI	Yes	9	100.0%	14	70.0%	3.404	*P* = 0.137
	No	0	0.0%	6	30.0%		
Preoperative Rectocele in MRI	Grade I	3	33.33%	12	60.0%	1.768	*P* = 0.245
	Grade II	6	66.67%	8	40.0%		
Preoperative Intussusception in MRI	Yes	9	100.0%	3	15.0%	18.488	***P*** < 0.001^*^
	No	0	0.0%	17	85.0%		

^a^Chi-square test

^*^Statistically significant difference

### Age

Patients with persistent symptoms were significantly older than those who improved. The mean age was 55.67 ± 7.05 years versus 37.55 ± 6.38 years in the improved group. Independent t-test confirmed this difference was statistically significant with a mean difference of 18.12 years (95% CI: 12.96 to 23.54; *p* < 0.001).

### ARJ descent

On preoperative MRI defecography, the mean anorectal junction (ARJ) descent was greater in the persistent symptoms group (7.50 ± 1.89 cm) than in the improved group (5.65 ± 0.62 cm). Independent t-test showed this difference was statistically significant, with a mean difference of 1.85 cm (95% CI: 0.39 to 3.31; *p* = 0.019).

### Preoperative rectocele

Rectocele was present in all patients. In the persistent symptoms group (*n* = 9), 33.3% were grade I and 66.7% grade II, compared to 60.0% grade I and 40.0% grade II in the improved group (*n* = 20). Chi-square test showed no significant difference in grade distribution (*p* = 0.55). Mean rectocele size was similar between both groups (2.50 ± 0.90 cm vs. 2.50 ± 0.92 cm), with no significant difference by independent t-test (*p* = 1.00).

### Preoperative intussusception

Chi-square test demonstrated a significantly higher prevalence of intussusception in patients with persistent symptoms compared to those who improved (*p* < 0.001). All patients in the persistent symptoms group exhibited intussusception, whereas only 3 of 20 patients (15.0%) in the improved group were affected.

### Preoperative perineal body dimensions

Independent t-tests used to compare perineal body dimensions in MRI defecography. Patients with persistent symptoms had smaller perineal body dimensions compared to the improved group. Cranio-caudal length was 16.67 ± 1.04 mm versus 18.32 ± 0.72 mm (*p* < 0.001), and antero-posterior length was 14.70 ± 0.15 mm versus 15.74 ± 0.62 mm (*p* < 0.001).

## Discussion

Perineal descent syndrome has been frequently reported in patients presenting with chronic constipation [18]. PDS was first described by Parks et al. in 1966 as an increased ballooning of the perineum below the bony pelvic outlet in association with obstructed defecation symptoms [3]. The anorectal junction is controlled by activity of both internal and external anal sphincter and muscles of pelvic floor [19]. The relationship between PD and ODS is grounded in physiology of defecatory process, in particular the mechanics of the Valsalva maneuver. In normal individuals, Laplace’s law explains how this maneuver decreases abdominal cavity volume and increases intra-abdominal pressure, promoting stool evacuation. In contrast, in people with increased PD, the Valsalva maneuver may simply reshape the abdominal cavity without producing the necessary abdominal and pelvic pressure for effective defecation [11]. Most of the currently proposed surgical procedures focus on correction of rectal intussusception and/or rectocele [8, 9]. D’Amico and Angriman argued that complete recovery from the perineal descent syndrome was difficult to be expected so treatment should focus on improving symptoms [20].

Our results demonstrated significant improvements in both clinical symptoms and objective measurements of perineal descent following surgery. However, because this was an uncontrolled cohort study without postoperative MRI confirmation, these findings should be interpreted as associational rather than causal. Furthermore, the small absolute reduction in perineal descent measured by perineometry (0.64 cm) should be interpreted with caution, given the lack of imaging validation and the potential for measurement bias. Also, we acknowledge another important limitation that CCCS are known to correlate poorly with anatomical measurements. The mean CCCS, a key measure of obstructed defecation, decreased significantly from 18.00 ± 3.21 preoperatively to 12.69 ± 4.65 postoperatively (*p* < 0.001), indicating an improvement in obstructed defecation severity. These findings are in agreement with Renzi et al. In 2016, who reported similar symptom improvement following surgical correction of perineal descent using a porcine dermal implant [11]. In 2025, Renzi et al. also demonstrated that adding the transverse perineal procedure to stapled trans-anal rectal resection (STARR) in patients complaining of ODS, internal rectal prolapse, and pathological perineal descent effectively corrected perineal descent and improved long-term outcomes without more morbidity or hospital stay [21]. In the present study, Postoperative extent of perineal descent measured by perineometer was significantly reduced. These findings suggest that the TPS procedure may contribute to the restoration of perineal support, Although this mechanistic interpretation is limited by the absence of postoperative MRI defecography. These results are consistent with those of Renzi et al., in which the mean postoperative perineal descent measured on MRI defecography (2.15 ± 0.8 cm) was significantly lower than the preoperative values (4.16 ± 14.6 cm) [11].

A significant increase in postoperative intrarectal pressure during push was observed, rising from a preoperative value of 34.34 ± 6.03 mmHg to 41.17 ± 5.85 mmHg postoperatively (*p* < 0.001). Intrarectal pressure during push is highly dependent on patient effort, cooperation, and understanding of the maneuver. Despite standardized instructions, we cannot exclude the possibility of measurement bias. This finding is consistent with Renzi et al., who reported a similar increase in mean intrarectal pressure following surgical correction of perineal descent, from 45.9 ± 12.8 mmHg preoperatively to 69.4 ± 11.1 mmHg postoperatively [11]. These results support the hypothesis that the TPS procedure may reinforce the perineal body and transverse perineal muscle, potentially contributing to a significant increase in intrarectal pressure.

In this study, 9 of 29 patients (31.03%) continued to experience symptoms after surgery. This subgroup was older, with a mean age of 55.67 ± 7.05 years, compared to 37.55 ± 6.38 years in the improved group, suggesting that age may be associated with a lower likelihood of symptom resolution. We report this as an observed association only. It is not possible from this study to conclude that age causes lower likelihood of symptom resolution, as older patients may have longer disease duration, more advanced pelvic floor degeneration, greater tissue laxity, or other unmeasured confounders. These findings are generally consistent with those of Murad-Regadas et al., who observed that posterior pelvic floor dysfunction, such as rectocele with rectal intussusception, was more frequent in women aged above 50 years of age [22]. Similarly, Wijffels et al. reported a tendency for internal rectal prolapse to increase with advancing age [23].

The prevalence of intussusception was also more in the persistent symptoms group (100%) compared to the improved group (15%), which may indicate a potential association between intussusception and less favorable outcomes following surgery for obstructed defecation syndrome. This observation aligns with previous reports by Renzi et al., who noted that intussusception persisted in some patients after surgical intervention [11].

In Comparison with other treatment modalities, The TPS procedure targets a different pathophysiological mechanism (perineal laxity) compared to STARR (which resects intussusception) or laparoscopic ventral mesh rectopexy (which suspend rectal prolapse). TPS is not currently recommended in major colorectal guidelines (e.g., ASCRS, ESCP) and should be considered an investigational procedure. Pelvic floor physiotherapy remains the first-line treatment for mild to moderate PD, and surgery should be reserved for refractory cases with anatomical evidence of pathological descent.

This study assessed the association between perineal descent and patient factors including age, rectal intussusception, and rectocele grade. Patients Above 40 years had a greater mean anorectal junction (ARJ) descent compared with patients aged 40 years or younger (6.82 ± 1.78 cm vs. 5.66 ± 0.66 cm; *P* = 0.035). This is consistent with Baek et al., who reported that perineal descent tends to increase with advancing age [24]. Similarly, Murad-Regadas et al. observed that posterior pelvic floor dysfunction, including significant rectocele and rectal intussusception, was more frequently associated with older age [21]. Perineal descent was also significantly greater in patients with intussusception (7.20 ± 1.70 cm) than in those without (5.53 ± 0.60 cm; *P* = 0.006). This finding aligns with Baek et al., who reported that perineal descent was significantly greater in patients with intussusception than in those without [24]. In this study, rectocele grade did not appear to significantly affect the extent of perineal descent. Patients with grade II rectoceles had a mean descent of 6.24 ± 1.34 cm, only slightly higher than the 6.20 ± 1.56 cm observed in patients with grade I rectoceles (*P* = 0.937). These results differ from Baek et al., who reported that larger rectoceles were associated with greater perineal descent [24].

Our study found that extent of perineal descent measurements obtained with a perineometer were lower than those measured by MRI defecography, with a statistically significant difference between the two modalities. The mean descent measured by perineometer was 2.29 ± 0.55 cm, whereas MRI defecography showed a mean descent of 6.22 ± 1.43 cm (*P* < 0.001). These results are consistent with those of G.J. Oettle et al., who reported that the use of perineometer underestimated pelvic floor movement by 60%, with a mean descent of 1.2 cm compared to 2.9 cm on radiographic assessment (*P* < 0.001) [25].

Although the perineometer provides a radiation-free and non-invasive method for assessing perineal descent, it appears less sensitive than defecographic techniques. One limitation is that the perineometer measures descent based on the anal verge rather than the anorectal angle, which can change during defecation due to anal canal shortening. Additional sources of variability include differences in soft tissue thickness, particularly in obese patients, and challenges in consistently positioning the device due to adjacent anatomical structures. In contrast, MRI defecography allows direct visualization of pelvic floor dynamics and the actual evacuation of contrast material, providing more accurate and reproducible measurements during simulated defecation. Therefore, while the perineometer may be useful as a clinical screening tool, defecographic imaging is likely more reliable for precise quantification of perineal descent.

## Conclusion and recommendations

Transverse perineal support procedure may be an effective option for selected patients with ODS and pathological PD. Outcomes were less favorable in patients with rectal intussusception, older age (observed as an association rather than a causal factor) or smaller perineal body dimensions, underscoring the importance of careful patient selection and individualized surgical planning. Given the small sample size and the absence of a control group or postoperative MRI confirmation, these findings should be considered with caution as associational rather than causal. Further studies with larger sample sizes, control groups, and longer follow-up are warranted to confirm durability and functional outcomes. Also, proper detailed assessment and tailored treatment plan for each patient are the key for better outcome.

### Strengths and limitations

This study has certain limitations. The small, feasibility-based sample size limits precision and generalizability. The short follow-up (six months) precludes assessment of long-term outcomes, and the absence of a control group limits causal inference. The lack of postoperative MRI defecography prevented evaluation of morphological changes, weakening mechanistic conclusions. Numerous subgroup analyses increase the risk of type I and type II errors and should be considered hypothesis-generating only. Despite these limitations, the prospective design and use of validated outcome measures are strengths of this study. This study could be a foundation for the development of more studies for more detailed evaluation of transverse perineal support procedure in ODS with pathological perineal descent.

## Data Availability

The datasets used and/or analyzed during the current study are available from the corresponding author on reasonable request.

## Abbreviations

ARM: Anorectal manometry
BMI: Body Mass Index
CCCS: Cleveland Clinic Constipation Score
CI: Confidence Interval
IQR: InterQuartile Range
ODS: Obstructed Defecation Syndrome
PD: Perineal Descent
PISQ-12: Pelvic organ prolapse/Urinary Incontinence Sexual Questionnaire
SD: Standard Deviations
STARR: stapled trans-anal rectal resection
TPS: Transverse Perineal Support
VAS: Visual Analogue Scale
